# The Mutational Landscape of SARS-CoV-2 Variants of Concern Recovered From Egyptian Patients in 2021

**DOI:** 10.3389/fmicb.2022.923137

**Published:** 2022-07-07

**Authors:** Mohamed G. Seadawy, Reem Binsuwaidan, Badriyah Alotaibi, Thanaa A. El-Masry, Bassem E. El-Harty, Ahmed F. Gad, Walid F. Elkhatib, Maisra M. El-Bouseary

**Affiliations:** ^1^Biological Prevention Department, Egypt Army, Cairo, Egypt; ^2^Department of Pharmaceutical Sciences, College of Pharmacy, Princess Nourah Bint Abdulrahman University, Riyadh, Saudi Arabia; ^3^Department of Pharmacology and Toxicology, Faculty of Pharmacy, Tanta University, Tanta, Egypt; ^4^Department of Microbiology and Immunology, Faculty of Pharmacy, Ain Shams University, Cairo, Egypt; ^5^Department of Microbiology and Immunology, Faculty of Pharmacy, Galala University, New Galala City, Egypt; ^6^Department of Pharmaceutical Microbiology, Faculty of Pharmacy, Tanta University, Tanta, Egypt

**Keywords:** SARS-CoV-2, next generation sequencing, delta variant, alpha variant, variant of concern, phylogenetic analysis, Egypt

## Abstract

In December 2019, a mysterious viral pneumonia first developed in Wuhan, China, resulting in a huge number of fatal cases. This pneumonia, which was named COVID-19, was attributed to a novel coronavirus, SARS-CoV-2. The emerging SARS-CoV-2 mutations pose the greatest risk to human health because they could result in an increase in the COVID-19 severity or the failure of current vaccines. One of these notable mutations is the SARS-CoV-2 Delta variant (B.1.617) that was first detected in India and has rapidly expanded to 115 countries worldwide. Consequently, in this study, we performed next-generation sequencing and phylogenetic analysis of SARS-CoV-2 during the third wave of the pandemic to determine the SARS-CoV-2 variants of concern (VOC) prevalence in Egypt. We observed several mutational patterns, revealing that SARS-CoV-2 evolution has expanded in Egypt with a considerable increase in the number of VOC. Therefore, the Egyptian authorities should take an appropriate approach to investigate the compatibility of already employed vaccines with this VOC and to examine the efficacy of the existing therapeutic regimen against new SARS-CoV-2 variants.

## Introduction

In December 2019, a mysterious viral pneumonia first developed in Wuhan, China, resulting in a huge number of fatal cases (Wu et al., 2020a). This pneumonia, which was named COVID-19, was attributed to a novel coronavirus, severe acute respiratory syndrome coronavirus 2 (SARS-CoV-2; Uddin et al., 2020). Due to its incredible rapid expansion, within a few months, the outbreak had reached 215 countries, including Africa, and accounted for more than 38 million infected people around the world (Lin et al., 2020; Pillay et al., 2020). Almost a year later, SARS-CoV-2 was blamed for around 4,757,626 deaths worldwide, according to a world health organization database (World Health Organization, 2021). On March 14, 2020, Egypt declared a state of emergency after the first COVID-19 confirmed cases were reported on a Nile cruise ship and accounted for the prompt Egyptian governmental preventive measures (Kandeil et al., 2020). As of September 26, 2021, Egypt had confirmed 300,945 cases in total, including 17,149 deaths (case-fatality rate, 5.7%), according to the Johns Hopkins Center update (Johns Hopkins Center, 2021). Egypt ranks seventh globally in case fatality rates with COVID-19, despite its low incidence and morbidity rates (Saied et al., 2021).

According to the phylogenetic analysis, coronaviruses are classified into four genera: alpha-, beta-, gamma-, and delta-coronaviruses, within the family Coronaviridae of the Nidovirales order. Consequently, SARS-CoV-2 is classified as a beta-coronavirus. The beta-coronaviruses are further classified into four lineages (A to D; Peiris et al., 2003). At least seven main clades (as identified by the GISAID database) have been detected in the sequenced genomes of SARS-CoV-2 so far. In addition, it is well known that coronavirus sequences undergo continuous changes because of recurrent mutations, and it is still unclear whether these mutations are due to an ongoing adaptation or genetic drift. There have also been no reports of distinct evolutionary patterns in SARS-CoV-2 genomes in Egypt (Van Dorp et al., 2020; Chiara et al., 2021; Mandal et al., 2021). Additionally, such emerging mutations pose the greatest risk to human health because they could result in change in COVID-19 severity or the reduction of the effect of the vaccines currently in development. The fundamental reason for this is that viral signals may be able to overcome immune protection established by a previous infection or immunization (Collier et al., 2021; Hamed et al., 2021).

The mutations encoding the S-protein are the most prominent, with a small number of them occurring in the S protein’s receptor-binding motifs, which are important for viral entrance *via* binding to human angiotensin-converting enzyme 2 (ACE 2) receptors (Mittal et al., 2020). One of these notable mutations is the Delta mutation, also known as B.1.617, which was first discovered in India in September 2020 and has since been extended to 115 nations around the world (Li et al., 2021). It has a group of spike protein mutations in which S_E484Q and S_L452R mutations were first seen together. The SARS-CoV-2 has been demonstrated to be affected by each of these mutations separately, but the cumulative effect of these mutations is unidentified (Yadav et al., 2021).

Furthermore, phylogenetic studies of SARS-CoV-2 and virus mutation sites are necessary for tracking down the source of infection and helping with infection control through sequence-based mRNA vaccine development (Freyn et al., 2020; Ma et al., 2020; Pillay et al., 2020). Thus, close monitoring of the pattern of changes in SARS-CoV-2 mutations is urgently required. Reviewing the literature, few studies tried to clearly investigate the evolutionary patterns of SARS-CoV-2 mutations of 2021 strains. Therefore, this study aimed to gain a deep elucidation of the molecular epidemiology of the outbreak in Egypt through a phylogenetic analysis of the genomic sequences of SARS-CoV-2 isolated from COVID-19 Egyptian patients in 2021.

## Materials and Methods

### Sample Collection

Nasal swabs were collected from patients hospitalized at the Army Hospital between August and September 2021. A total of 172 patients chosen to participate in this study were suspected of being COVID-19 positive and exhibiting infection symptoms. Before taking part in the study, all subjects gave their informed consent to be included. The study was carried out in line with the Helsinki Declaration and after approval by the PNU Institutional Review Board (IRB number: 20–0457). The samples were transported to the biological prevention department in a viral transport medium (Cat. No. A48498) at 4°C, in accordance with strict biosafety regulations.

### rRT-PCR Confirmatory Test

The Qiaamp^®^ Viral RNA Mini Kit (Qiagen, Germany) was Used for the Extraction of Viral RNA From Each Sample. Following the Purification of Viral RNA, Semi-Quantitative Amplification of the Extracted RNA was Performed by the Viasure^®^ SARS-CoV-2 RT-PCR Detection Kit (ref. *VS*-NCO212H) Using the Ariamx RT-PCR Equipment (Agilent, US).

### Next Generation Sequencing

The AviSeq COV19 NGS Library Prep kit (Ref. AVG202096) was used to prepare the libraries according to the manufacturer’s instructions. Next-generation sequencing was performed using the Illumina iSeq 100^®^ System (Illumina, US). The Qubit^™^ RNA HS 100 Assay Kit (Cat. No. Q32852) was used to quantify RNA on a Qubit 2.0 Fluorometer. Then, complementary DNA was made using RT Primer Mix DP (BATCH No. 020402C). In addition, the whole SARS-CoV-2 genome was amplified using a multiplex PCR process. In order to eliminate any non-specific PCR products, digestion was performed. The DNA HS 100 Assay Kit (Cat.No.Q32852) was used to evaluate the quality of the library preparation.

### Genome Assembly

Next-generation sequencing was conducted on 172 isolated isolates. FASTA files were generated using the online tool DECOV^™^.^1^ The validated sequences, novel ones, were submitted to the NCBI Virus GenBank and received accession numbers (see Supplementary Table S1).

### Data Analysis

For viral genome alignment, quality inspection, clade determination, and phylogenetic studies, the online tool “Nextclade” was utilized.^2^ The Burrows-Wheeler Aligner (BWA) software was used to align the genome sequences. The BLAST tool was used to locate nucleotide and amino acid sequences that were similar (see Supplementary Table S1).

### Phylogenetic Analysis

Understanding the development and evolution of SARS-CoV-2 necessitates the use of phylogenetic trees. Molecular Evolutionary Genetics Analysis v.7.0 (MEGA7) software was used to conduct evolutionary studies (Kumar et al., 2016). The phylogenetic tree was constructed by using the Maximum Likelihood approach based on the Tamura-Nei model. Next to the branches is the percentage of trees in which the related taxa were grouped together in the bootstrap (100 repetitions). The heuristic tree involving 65 nucleotide sequences was created by applying the Neighbor-Join and BioNJ algorithms to a matrix of pairwise distances calculated using the Maximum Composite Likelihood (MCL) method, and then choosing the topologies with the highest log likelihood ratio. In order to visualize and edit the constructed ML trees, the “Interactive Tree Of Life” (iTOL v.5) online tool was used (Letunic and Bork, 2021).^3^

## Results

### The Data of Study Population

In the current study, 172 positive COVID-19 patients were selected based on clinical diagnosis, 130 (75.6%) males and 42 (24.4%) females, with an average age of 37 (Table 1).

**Table 1:** The distribution of female and male patients among various age groups.

**Patient gender**	**Age group**							
	**≤ 20**	**21–30**	**31–40**	**41–50**	**51–60**	**61–70**	**>70**	**Total**
**Female**	0	10	11	8	6	4	3	42
**Male**	2	43	38	19	12	10	6	130
**Total**	2	53	49	27	18	14	9	172

**Figure 1:**
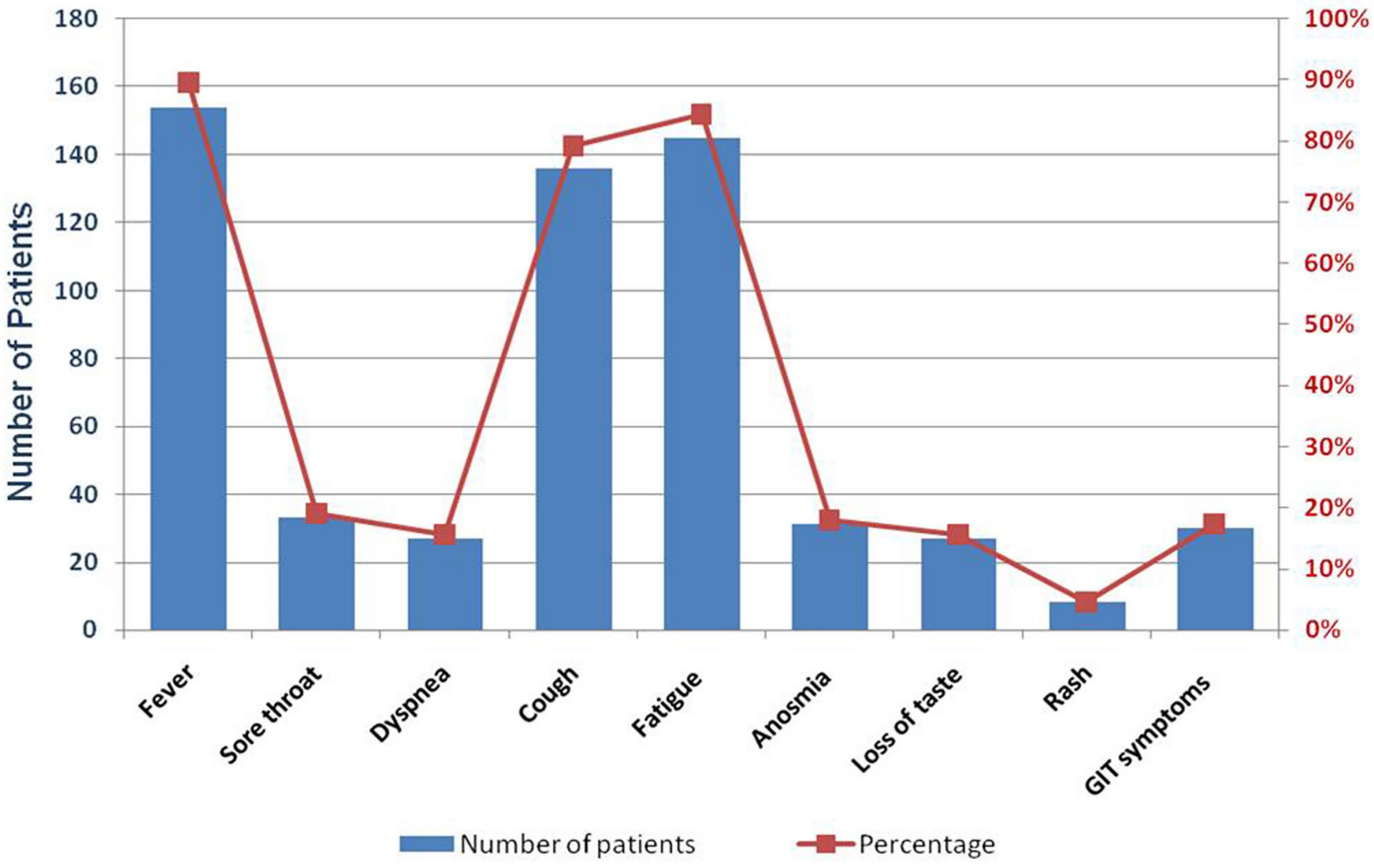
Bar chart showing both the number of patients exhibiting COVID-19 symptoms.

The clinical picture of the participants included in the present work is presented in Figure 1. Fever, fatigue, and cough were the predominant reported signs (90, 84, and 79%, respectively), then sore throat (19%), anosmia (18%), GIT symptoms such as nausea, vomiting, and diarrhea (17%), and loss of taste (16%). Only eight patients (5%) suffered from a rash.

### Confirmatory rRT-PCR Test

Based on rRT-PCR test results (Cq values ranging from 14 to 20), 172 samples were positive, proving SARS-CoV-2 viral infection.

### Sequence Data Analysis

Next generation sequencing (NGS) was performed on 172 strains. The lengths of the resulting genomic sequences were identical to the genome length of the Wuhan-Hu-1 reference strain (NC 045512.2). The generated FASTA files were uploaded to the NCBI Standard Nucleotide BLASTn Tool, and the Betacoronavirus database was selected before running.^4^ Several variants were detected among the tested isolates (*n* = 64). A total of 40 sequences were previously detected in Egypt (nucleotide identity = 100%) and had specific accession codes in the NCBI database. Twenty-four nucleotide sequences were novel and were submitted to NCBI GenBank to be provided with certain accession numbers.

**Figure 2:**
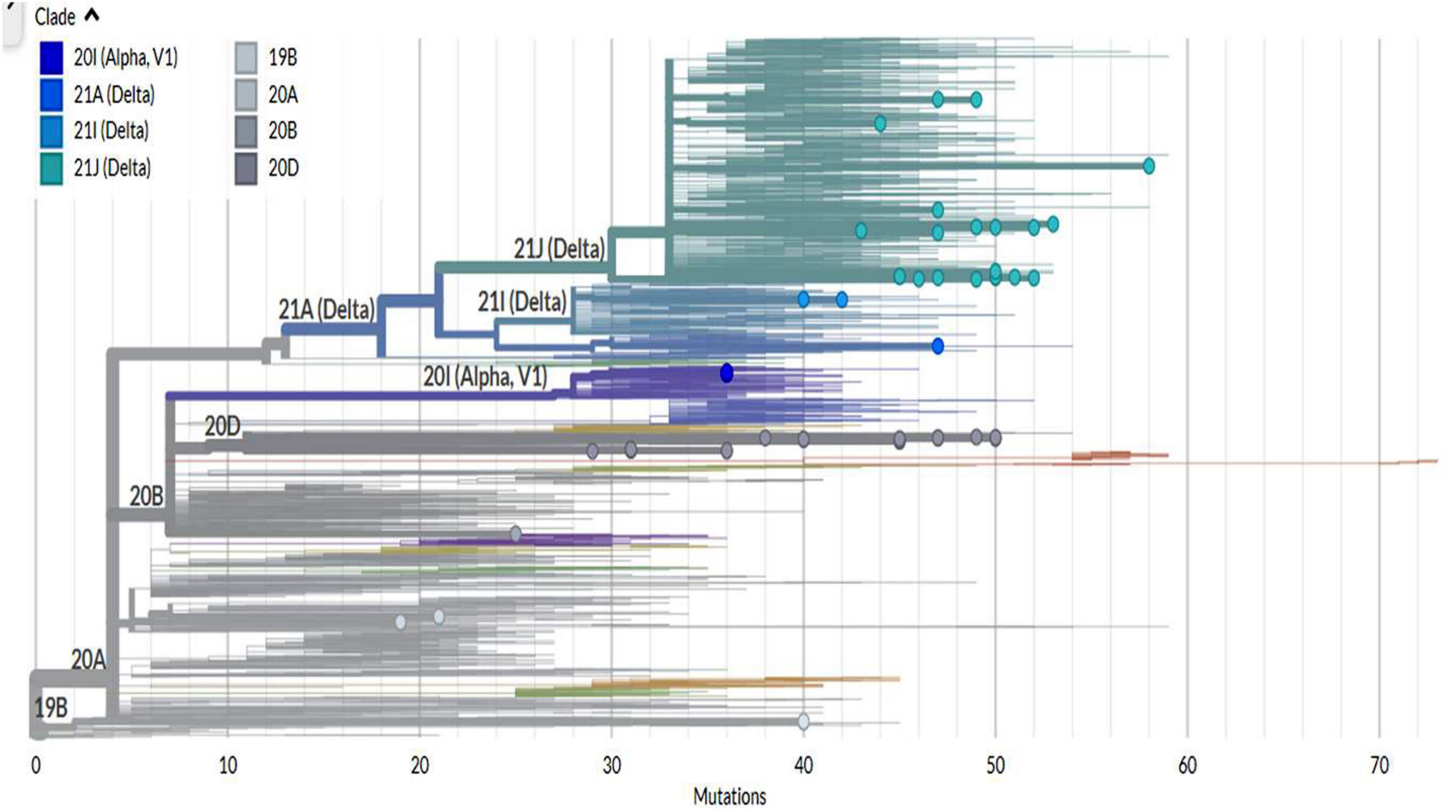
Rectangular phylogenetic tree showing the distribution of sequences among different clades (https://clades.nextstrain.org/tree).

The Nextclade Tool was used for the detection of genetic mutations in the SARS-CoV-2 genome, including S, E, M, N, ORF1a, ORF1b, ORF3a, ORF6, ORF7a, ORF7b, ORF8, and ORF9b (see Supplementary Table S1). The sequences under investigation were clustered into eight distinct clades, including 19B, 20A, 20B, 20D, 20I (Alpha variant), 21A (Delta), 21I (Delta), and 21 J (Delta) as shown in Figure 2.

**Figure 3:**
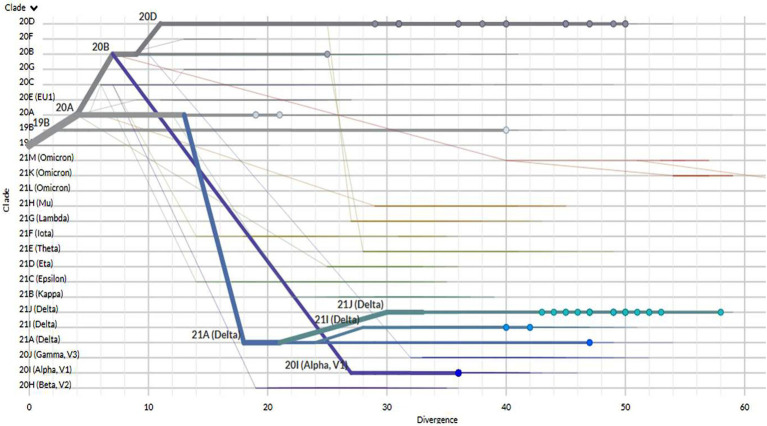
The divergence of tested SARS-CoV-2 genomes in comparison to the reference strain Wuhan-Hu-1.

A rectangular phylogenetic tree constructed by the online Nextclade Tool revealed the most common clades were 20D (34.4%) and 21 J (32.8%), followed by 20I (12.5%), 20A (9.4%), and 19B (4.7%). Further investigation to detect the evolution of SARS-CoV-2 was conducted by determination of the divergence of the tested sequences in relation to Wuhan-Hu-1/2019 (GenBank: MN908947; Figure 3).

As shown in Table 2, there were three sequences clustered as 19B with two mutations in the spike gene (H655Y- N501T). A single mutation, S_D614G, was reported in six sequences that were clustered as the 20A clade. The clade 20B was recorded in only one sequence with two mutations (D614G- N501T).

**Table 2:** Spike-gene mutations of the sequences involved in the study.

**Clade**	**Sequence Accession code**	**Number of patents**	**Quality**						**Ns Mut.**	**Spike-gene Mut**
			**N**	**M**	**P**	**C**	**S**	**F**		
**19B**	MZ087801	1	**√**	**√**	**x**	**√**	**√**	**√**	26	H655Y - N501T
	MZ087802	4	**√**	**√**	**x**	**√**	**√**	**√**	26	
	MZ087803	2	**√**	**√**	**x**	**√**	**√**	**√**	26	
**20A**	MZ042984	3	**√**	**√**	**√**	**√**	**√**	**√**	19	D614G
	MZ042986	5	**√**	**√**	**√**	**√**	**√**	**√**	19	
	MZ043021	1	**√**	**√**	**√**	**√**	**√**	**√**	21	
	MZ043022	5	**√**	**√**	**√**	**√**	**√**	**√**	21	
	MZ093207	1	**√**	**√**	**√**	**√**	**√**	**√**	19	
	MZ093208	2	**√**	**√**	**√**	**√**	**√**	**√**	19	
**20B**	MZ087804	2	**√**	**√**	**x**	**√**	**√**	**√**	23	D614G - N501T
**20D**	MZ042981	4	**√**	**√**	**x**	**√**	**√**	**√**	31	D614G - L452R - Q677H
	MZ042994	12	**√**	**√**	**x**	**√**	**√**	**√**	29	
	MZ042999	8	**√**	**√**	**x**	**√**	**√**	**√**	29	
	MZ043000	6	**√**	**√**	**x**	**√**	**√**	**√**	29	
	MZ043001	2	**√**	**√**	**x**	**√**	**√**	**√**	29	
	MZ043004	4	**√**	**√**	**x**	**√**	**√**	**√**	29	
	MZ043018	5	**√**	**√**	**x**	**√**	**√**	**√**	32	
	MZ043024	3	**√**	**√**	**x**	**√**	**√**	**√**	32	
	MZ043006	2	**√**	**√**	**√**	**√**	**√**	**√**	28	A899S - D614G - L452R - R346S
	MZ043016	3	**√**	**√**	**x**	**√**	**√**	**√**	27	D614G - L452R - Q677H - S12F
	MZ043023	5	**√**	**√**	**x**	**√**	**√**	**√**	27	
	MZ087794	1	**√**	**√**	**√**	**√**	**√**	**√**	37	A871S - A899S - D614G - Q677H
	MZ087795	11	**√**	**√**	**√**	**√**	**√**	**√**	37	
	MZ087796	1	**√**	**√**	**√**	**√**	**√**	**√**	32	A899S - D614G - L452R - Q677H - R346S - S12F - T572I - W152R
	MZ087798	1	**√**	**√**	**√**	**√**	**√**	**√**	32	
	MZ087800	2	**√**	**√**	**√**	**√**	**√**	**√**	37	A899S - D614G - L452R - R346S - W152R
	MZ087816	5	**√**	**√**	**√**	**√**	**√**	**√**	37	
	MZ087807	2	**√**	**√**	**x**	**√**	**√**	**√**	34	A222V- D614G - L452R - Q677H
	MZ087808	4	**√**	**√**	**x**	**√**	**√**	**√**	34	
	MZ087819	3	**√**	**√**	**√**	**√**	**√**	**√**	37	A871S - A899S - D614G - L452R - Q677H - R346S - S12F - W152R
	MZ087820	1	**√**	**√**	**√**	**√**	**√**	**√**	37	
	MZ093192	2	**√**	**√**	**x**	**√**	**√**	**√**	29	D614G - L452R - Q613H - Q677H
**20I (Alpha, V1)**	MZ042987	2	**√**	**√**	**√**	**√**	**√**	**√**	30	A570D - D614G - D1118H - N501Y - P681H - T716I
	MZ042988	8	**√**	**√**	**√**	**√**	**√**	**√**	30	
	MZ042989	5	**√**	**√**	**√**	**√**	**√**	**√**	30	
	MZ042990	8	**√**	**√**	**√**	**√**	**√**	**√**	30	
	MZ093203	4	**√**	**√**	**√**	**√**	**√**	**√**	30	
	MZ093204	2	**√**	**√**	**√**	**√**	**√**	**√**	30	
	MZ093205	2	**√**	**√**	**√**	**√**	**√**	**√**	30	
	MZ093195	3	**√**	**√**	**√**	**√**	**√**	**√**	34	A570D - D614G - D1118H - N501Y - P681H - S982A - T716I
**21A (Delta)**	OK354428	1	**√**	**√**	**x**	**√**	**√**	**√**	36	D614G - D950N - L452R - P681R - R158G - T19R - T478K
**21I (Delta)**	OK354427	1	**√**	**√**	**√**	**√**	**√**	**√**	32	A222V - D614G - D950N - L452R - P681R - R158G - T19R - T478K - V1264L
	OK354429	1	**√**	**√**	**√**	**√**	**√**	**√**	35	
**21 J (Delta)**	OK354409	1	**√**	**√**	**√**	**√**	**√**	**√**	42	D614G - D950N - L452R - P681R - R158G - T19R - T95I - T478K	
	OK354411	1	**√**	**√**	**√**	**√**	**√**	**√**	37			
	OK354424	1	**√**	**√**	**√**	**√**	**√**	**√**	39		
	OK354410	1	**√**	**√**	**√**	**√**	**√**	**√**	39	D614G - D950N - L452R - P681R - T19R - T95I - T478K
	OK354416	1	**√**	**√**	**x**	**√**	**√**	**√**	45	
	OK354419	1	**√**	**√**	**√**	**√**	**√**	**√**	41	
	OK354412	2	**√**	**√**	**√**	**√**	**√**	**√**	37	D614G - D950N - L452R - P681R - R158G - T19R - T478K
	OK354415	1	**√**	**√**	**√**	**√**	**√**	**√**	39	
	OK354417	1	**√**	**√**	**x**	**√**	**√**	**√**	38	
	OK354413	1	**√**	**√**	**x**	**√**	**√**	**√**	40	D614G - D950N - G1167V - L452R - P681R - R158G - T19R - T478K
	OK354414	1	**√**	**√**	**√**	**√**	**√**	**√**	51	D614G - D950N - L1265F - L452R - P681R - Q677H - R158G - T19R - T95I - T478K
	OK354418	1	**√**	**√**	**√**	**√**	**√**	**√**	38	D614G - D178H - D950N - L452R - P681R - R158G - T19R - T95I - T478K
	OK354420	1	**√**	**√**	**√**	**√**	**√**	**√**	41	D614G - D950N - E484Q - L452R - P681R - R158G - T19R - T478K - T572I
	OK354421	1	**√**	**√**	**x**	**√**	**√**	**√**	38	D614G - D950N - G1124R - L452R - P681R - R158G - T19R - T478K
	OK354422	1	**√**	**√**	**√**	**√**	**√**	**√**	38	D614G - D950N - L452R - P681R - R158G - T19R
	OK354423	1	**√**	**√**	**√**	**√**	**√**	**√**	41	D614G - D950N - L452R - P681R - Q677H - R158G - T19R - T478K
	OK354425	1	**√**	**√**	**x**	**√**	**x**	**√**	43	D614G - D950N - G181V - L452R - P681R - R158G - T19R - T478K
	OK354426	1	**√**	**√**	**√**	**√**	**√**	**√**	42	D614G - D950N - L452R - P681R - R158G - T19R - T478K - T572I
	OK354430	1	**√**	**√**	**x**	**√**	**√**	**√**	44	D614G - D950N - L452R - P681R - T19R - T478K - T572I
	OK354431	1	**√**	**√**	**√**	**√**	**√**	**√**	41	D614G - D950N - L452R - P681R - Q173H - R158G - T19R - T478K
	OK354432	1	**√**	**√**	**x**	**√**	**√**	**√**	38	D178Y - D950N - L452R - P681R - R158G - T19R - T478K

N, Missing Data; M, Mixed Sites; P, Private Mutations; C, Mutation Clusters; F, Frame shifts; S, Stop codons; Ns Mut, Numbers of Mutations.

Moreover, 22 sequences were grouped into a 20D clade with variable spike protein mutations (Table 2). The S-gene mutations observed were A871S, A899S, A222V, D614G, L452R, Q677H, Q613H, R346S, S12F, T572I, and W152R.

However, 8 sequences were grouped into a 20I clade (Alpha, V1), revealing two patterns of spike protein mutations including (A570D- D614G- D1118H- N501Y- P681H- T716I) and (A570D- D614G- D1118H- N501Y- P681H- S982A- T716I) that were recorded in 7 sequences and in one sequence, respectively (Table 2).

The clade 21A (Delta) was reported in a single sequence (accession code OK354428). Furthermore, two sequences were clustered as clade 21I (Delta) with an S-gene mutational pattern (A222V- D614G- D950N- L452R- P681R- R158G- T19R- T478K- V1264L). Finally, a total of 21 sequences were clustered as 21 J (Delta) with 15 unique S-gene mutational patterns (Table 2). These Delta variants were considered the first India VOC Delta strains to be reported and characterized in Egypt in 2021.

**Figure 4:**
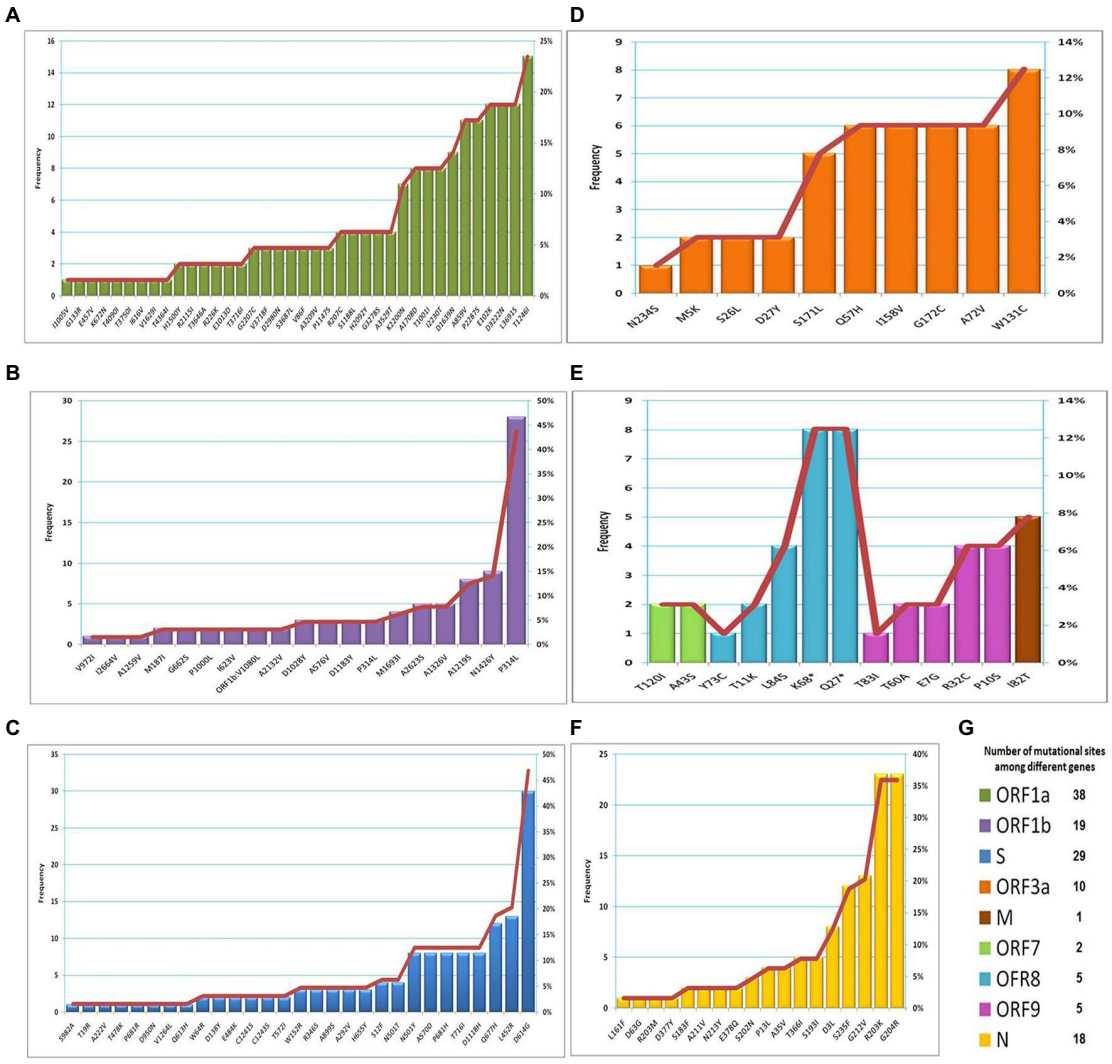
The frequency of mutations observed among different genes. **(A)** ORF1a; **(B)** ORF1b; **(C)** S; **(D)** ORF3a; **(E)** M, ORF7, ORF8 and ORF9; **(F)** N; **(G)** number of mutational sites distributed among genes.

The frequency of each mutation detected in recovered variants (n = 64) is presented in Figures 4A–F. The most predominant mutation was S: D614G (47%), followed by ORF1b: P314L (44%), N: G204R (36%), N: R203K (36%) and ORF1a: T1246I (23%). The most common mutation in the ORF3a gene was W131C (13%). The mutations recorded in the ORF7 gene were ORF7a: T120I (3%) and ORF7b: A43S (3%). Five mutations in the ORF8 gene were detected including K68* (13%), Q27* (13%), L84S (6%), T11K (3%) and Y73C (2%). Similarly, five mutations were detected in the OFR9b gene, including R32C (6%), P10S (6%), T60A (3%), E7G (3%), and T83I (2%). Only one mutation (I82T) was observed in the M gene with a frequency of 8%. The total number of mutational sites was 127, distributed among different genes (Figure 4G). The highest number of mutational sites were observed in the ORF1a gene (n = 38), followed by the S gene (*n* = 29), the ORF1b gene (*n* = 19), the N gene (*n* = 18) and the ORF3a gene (n = 10).

**Figure 5:**
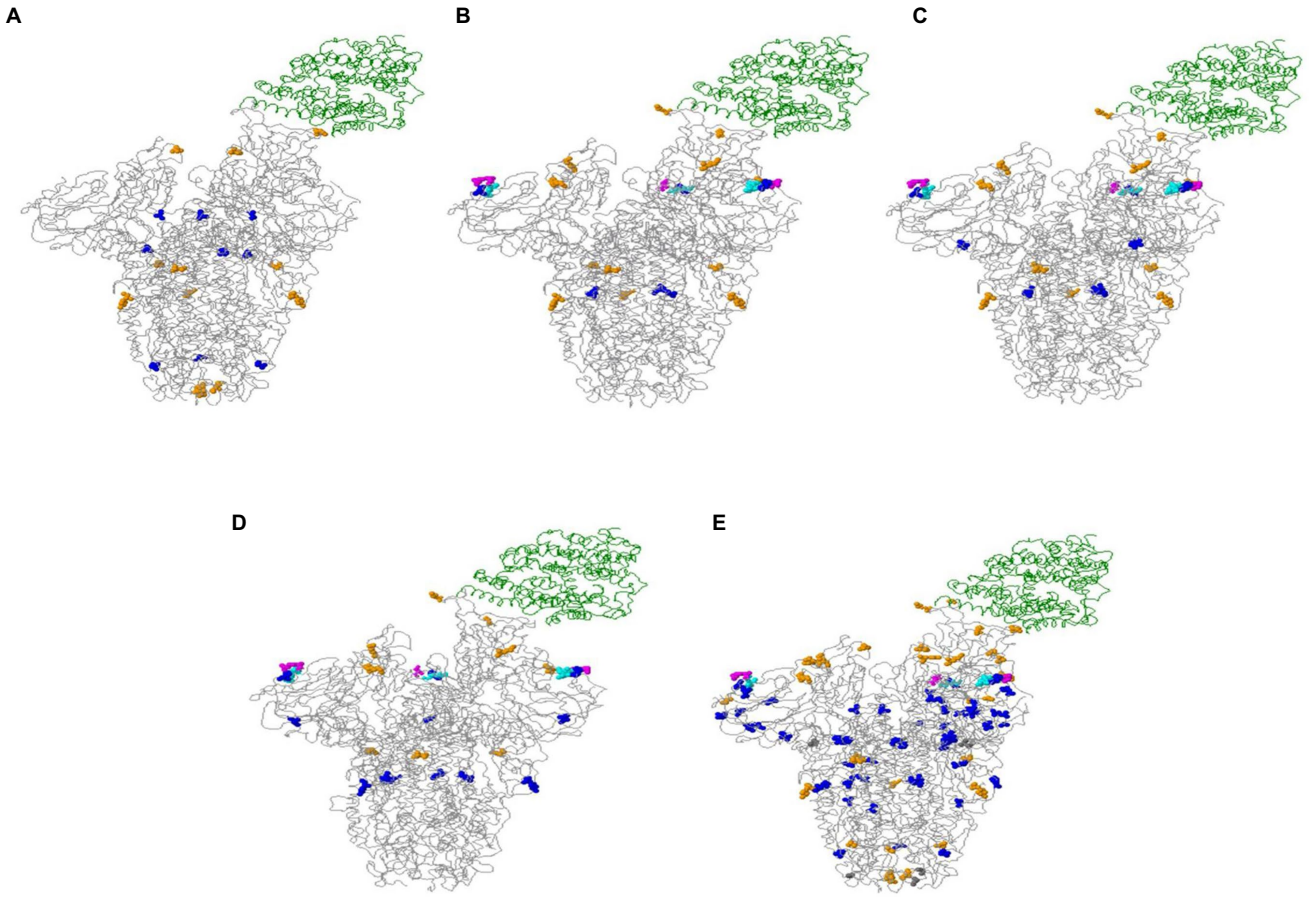
Spike glycoprotein associated with the host cell receptor ACE2 (green ribbon). **(A)** 20I (Alpha variant); **(B)** 21A (Delta variant); **(C)** 21I (Delta variant); **(D)** 21 J (Delta variant); and **(E)** overall changes in all sequences tested. Colored balls present amino acid variations: A222V, A570D, A871S, D138Y, D178H, D178Y, D614G, D950N, E156G, E484K, G181V, H655Y, Q173H, Q613H, Q677H, S982A, T95I, T572I, T716I, W64R in blue. A899S, D1118H, E484Q, L452R, N501T, N501Y, P681H, P681R, R346S, T478K, W152R in orange. A292V, G1124R in gray. F157del, R158del in turquoise. T19R in fuchsia.

The online Mutation Analysis tool, CoVsurver, was used for visualization of amino acid (aa) changes in the spike protein of the sequences under investigation.^5^ Several variations were recorded in the structure of the spike glycoprotein of the imported sequences (Figure 5). The variations displayed in the structure of VOC, such as 20I (Alpha), 21A (Delta), 21I (Delta), and 21 J (Delta) were 7, 9, 11, and 12, respectively.

Further characterization of tested genomes was performed to determine the PANGO lineage using the online tool “Pangolin COVID-19 Lineage Assigner”.^6^ The PANGO lineage of each sequence was presented in Table 3. Several variants of concern (VOC) are detected in the present study, including B.1.1.7 and B.1.617.2 + AY*, globally known as Alpha and Delta variants, respectively.

**Table 3:** PANGO lineage of the tested sequences.

**PANGO lineage**	**Sequences accession code**
A.28	MZ087801, MZ087802, MZ087803
B.1	MZ042984, MZ042986, MZ043021, MZ043022, MZ093207, MZ093208
B.1.1	MZ087804
C.36	MZ042981, MZ042994, MZ042999, MZ043000, MZ043001, MZ043004, MZ043016, MZ043018, MZ043023, MZ043024, MZ087807, MZ087808, MZ093192
C.36.3	MZ043006, MZ087794, MZ087795, MZ087796, MZ087798, MZ087819, MZ087820
C.38	MZ087800, MZ087816
B.1.1.7	MZ042987, MZ042988, MZ042989, MZ042990, MZ093195, MZ093203, MZ093204, MZ093205
B.1.617.2 + AY.62	OK354428
B.1.617.2 + AY.65	OK354427, OK354429
B.1.617.2 + AY.112	OK354411, OK354424
B.1.617.2 + AY.119.2	OK354409, OK354410, OK354416, OK354418, OK354419
B.1.617.2 + AY.122	OK354432
B.1.617.2 + AY.32	OK354420, OK354426
B.1.617.2 + AY.34.1	OK354414
B.1.617.2 + AY.39	OK354422
B.1.617.2 + AY.43	OK354415, OK354423
B.1.617.2 + AY.44	OK354412, OK354413, OK354417, OK354421, OK354425, OK354431
B.1.617.2 + AY.98.1	OK354430

### Phylogenetic Analysis

**Figure 6:**
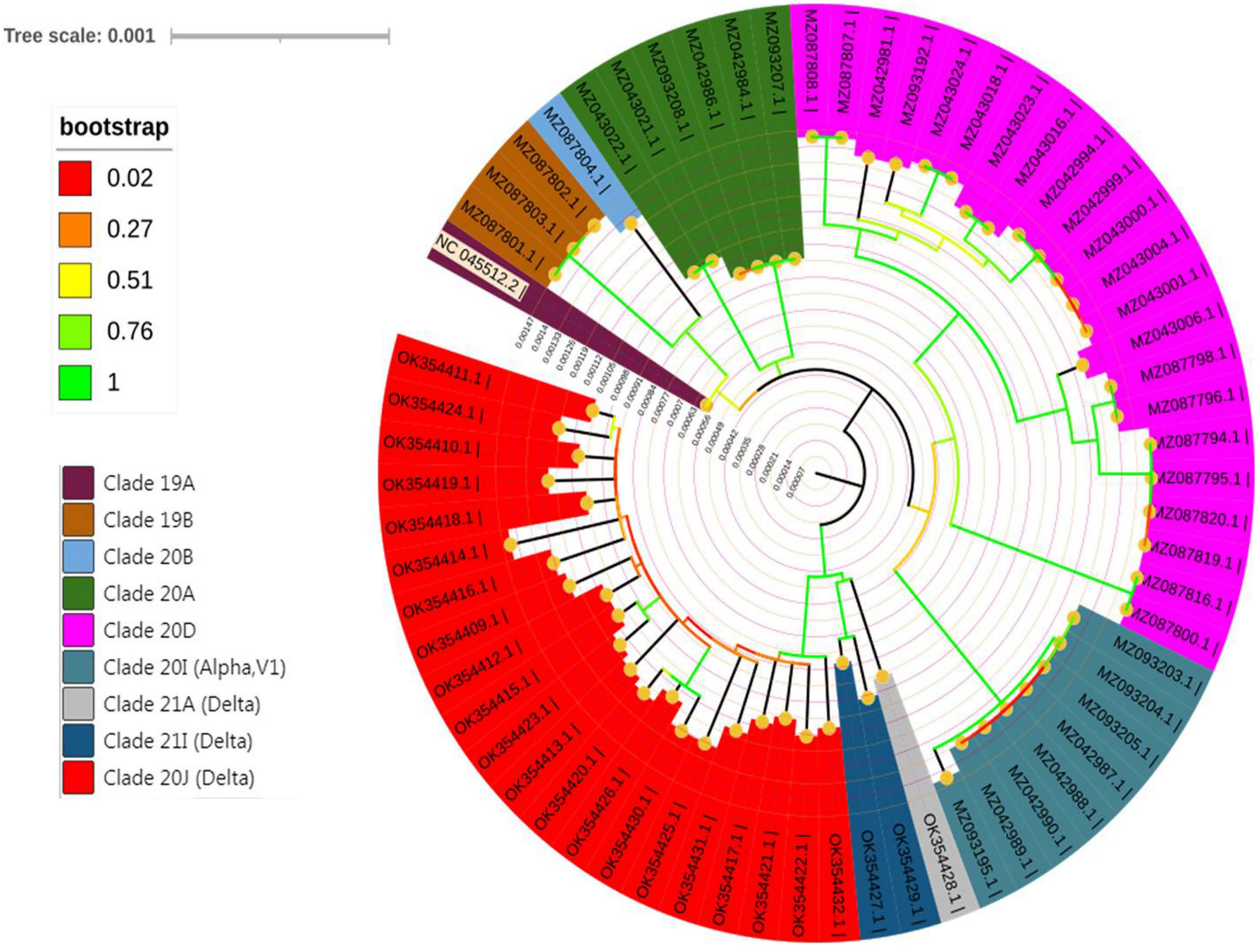
Molecular phylogenetic analysis by the Maximum Likelihood method.

The analysis involved 65 nucleotide sequences, including the recovered genomes in the current work and the reference strain Wuhan-Hu-1. Before running the analysis, the elimination of all positions with gaps and missing data was performed. There were a total of 29,267 positions in the testing dataset. The maximum likelihood phylogenetic analysis revealed that variants under investigation are clustered into distinct groups according to their evolutionary pattern, as shown in Figure 6.

## Discussion

We isolated SARS-CoV-2 from 172 patients admitted to the Army Hospital during the third wave of the pandemic between August and September 2021. We observed that males (75.6%) were more susceptible to COVID-19 infection than females (24.4%), with an approximate mean age of 37. This was in accordance with previous research conducted in Egypt during the first and second waves of COVID-19, which reported that those aged 40 to 60 were the most frequently infected (AboKresha et al., 2021; Alotaibi et al., 2022b). Furthermore, we recorded each patient’s clinical manifestations in order to contribute to the development of a more clear and comprehensive clinical presentation of COVID-19, which is still unclear due to the SARS-CoV-2 high mutational rate. We found that fever, fatigue, and cough were the predominant symptoms, while sore throat, loss of taste, anosmia, and GIT symptoms were reported but with less frequency. These observations are in line with the findings of previous investigations (Doghish et al., 2021; Ibrahim et al., 2021; Alotaibi et al., 2022b; Cascella et al., 2022).

Our results regarding the follow-up of SARS-CoV-2 mutations revealed that variants of concern, such as alpha and delta variants, are expanding. Samples have been clustered into 8 different subclades, including 19A, 19B, 20A, 20B, 20D, 21A, 21I, and 21 J. The majority of samples were from the 20D and 21 J subclades. SARS-CoV-2 has evolved into new variants and their frequency in Egypt and even globally has increased dramatically and extensively (Hodcroft et al., 2021; Seadawy et al., 2022). Several spike mutations have recently been identified in SARS-CoV-2 new variants, implying that COVID-19 could be more severe with a significant probability of immune escape (Harvey et al., 2021; Seadawy et al., 2022).

In the present study, we are the first to formally characterize the B.1.617.2 variant in Egypt. Moreover, we reported that these B.1.617.2 variants were clustered into three subclades, including 21A (n = 1), 21I (n = 2), and 21 J (n = 22) with A222V, D178H, D178Y, D614G, D950N, E484Q, G181V, G1124R, G1167V, L452R, L1265F, P681R, Q173H, Q677H, R158G, T19R, T478K, T95I, T572I, and V1264L amino acid mutations in S-gene. Since its first detection in a sequence from India, the B.1.617.2 variant has already spread throughout India and to at least 115 countries due to its fast rate of transmission and infection in comparison with other previously detected variants (Cherian et al., 2021; Li et al., 2021; Mlcochova et al., 2021). According to the World Health Organization, a virus belonging to the B.1.617 lineage has been recognized as a VOC. It appeared to have high transmission rates, with a rapid increase in prevalence documented in several nations. According to a structural analysis of B.1.617 receptor binding domain (RBD) mutations (L452R and E484Q, as well as P681R in the furin cleavage site), these variants may contribute to an increase in ACE2 binding and S1-S2 cleavage, resulting in improved disease transmission and possibly the ability to elude the immune protection activity from existing antibodies (Cherian et al., 2021; Outbreak. info, 2021).

However, vaccination is quite efficient in reducing COVID-19 severity and preventing death, but it is less effective in preventing infection by the delta and alpha VOC (Emary et al., 2021; Singanayagam et al., 2022). Several studies have been conducted to illustrate how SARS-CoV-2 S-protein mutations can affect the neutralization of antibodies. Garcia-Beltran et al. assessed the neutralization potency of 99 people who received one or two doses of either BNT162b2 or mRNA-1,273 vaccines against pseudoviruses, concluding that the potential for variants to evade vaccine responses emphasizes the necessity of broad protective intervention strategies against the pandemic’s rapid evolution (Garcia-Beltran et al., 2021). Also, Bates et al. reported that the new SARS-CoV-2 variants are resistant to inactivation by serum antibodies, suggesting lower protection against re-infection or an elevated risk of vaccine breakdown (Bates et al., 2021).

We also detected the existence of the alpha variant (B.1.1.7) among recovered samples, with a prevalence of 19.8%. Seven mutations in the S-gene were reported in the alpha variants, including A570D, D614G, D1118H, N501Y, P681H, T716I, and S982A. Moreover, the most prevalent clade among recovered samples was 20D (66%) with A871S, A222V, A899S, D614G, L452R, Q613H, Q677H, R346S, S12F, T572I, and W152R mutations in the spike protein. Furthermore, only one sequence was grouped into the 20B clade with N501T and D614G mutations in spike protein. Seven sequences with spike protein mutations (H655Y-N501T) were grouped together as the 19B clade. The clade 20A with the S_D614G mutation was reported in 17 samples. Our findings regarding the prevalence of different clades during the third wave of the COVID-19 pandemic are consistent with previous studies conducted in Egypt and worldwide (Davies et al., 2021; Doghish et al., 2021; Gravagnuolo et al., 2021; Alotaibi et al., 2022a; Hart et al., 2022; Lambrou et al., 2022; Seadawy et al., 2022).

Concerning mutations detected in the SARS-CoV-2 viral genome, the ORF1a, S, ORF1b, N, and ORF3a genes showed high-frequency mutations with 33 nonsynonymous mutations, including A570D, A1708D, D3L, D63G, D377Y, D614G, D950N, D1118H, G204R, G662S, I82T, I2230T, L452R, N501Y, P314L, P681H, P681R, P1000L, Q27*, R203K, R203M, S26L, S235F, S982A, T19R, T60A, T120I, T478K, T716I, T1001I, T3646A, and Y73C. The most common mutations in spike protein were D614G (47%), L452R (20%), Q677H (19%), and N501Y (13%). The S protein is involved in SARS-CoV-2’s entrance into the host cell. As a result, mutations in RBD result in higher transduction in a variety of cell types, increased resistance to proteolytic cleavage, increased contagiousness (Daniloski et al., 2020), and increased ACE2 affinity (Liu et al., 2021). Antibody neutralization was decreased by the L452R mutation, which boosted the virus’s capacity to infect (Liu et al., 2021). N501Y has been shown to affect the affinity of inactivating antibodies (Luan et al., 2021).

Both ORF1a and ORF1b encode for replicase polyproteins, which are necessary for viral RNA replication. As a result, antiviral drugs have focused on the SARS-CoV-2 conserved structure (Yin, 2020). RNA-dependent RNA polymerase (RdRp), which is positioned between ORF1a and ORF1b, has a functionally conserved area known as “high sequence similarity.” RdRp is a key component for application and translation and plays an essential role in the genetic detection of COVID-19 (Wu et al., 2020b). ORF3a is a unique short viral protein that is involved in its pathogenicity and is required for viral adaptation *in vitro*. Q57H and G251V are the most common synonymous mutations in ORF3a and could have a beneficial impact on viral adaptation (Kim et al., 2020).

SARS-CoV-2 mutations become more prevalent due to viral transmission expanding. Therefore, the governments are urged to improve surveillance and sequencing facilities. In addition, the systematic procedures should be followed to produce a clear illustration of SARS-CoV-2 variant transmission predominance based on regional context, as well as to spot unconventional events (Rubin, 2021). Vaccination seems to be the only measure to avoid a global epidemic from getting worse. A rigorous strategy should be implemented to assess vaccine effectiveness and to conduct post-licensing examinations to identify differences in resistance between the original virus and new variants (Malik et al., 2022).

The sequenced genomes of SARS-CoV-2 isolated from Egyptian patients allow the identification of circulating strains, especially VOC in Egypt. Our findings stress the urgent need for continuous follow-up of SARS-CoV-2 virus evolution and conducting real-time genomic surveillance and epidemiological studies to support public response to an existing pandemic. Furthermore, they are helpful to Egyptian healthcare decision-makers in tracking the prevalence of SARS-CoV-2 variants and understanding more about SARS-CoV-2 transmission and pathogenicity.

## Conclusion

In summary, we isolated SARS-CoV-2 from Egyptian COVID-19 patients during the third wave of the pandemic. The samples have been clustered into eight subclades: 19B, 20A, 20B, 20D, 20I (alpha, V1), 21A, 21I, and 21 J (delta). Several mutational patterns were observed in Egypt, revealing that SARS-CoV-2 evolution has expanded. The twenty-five isolates shown in the present study are the first fully characterized Delta variant (B.1.617.2) in Egypt. Therefore, the Egyptian Ministry of Health and Population as well as healthcare decision-makers must investigate the compatibility of already employed vaccinations with this VOC and examine the efficacy of the existing therapeutic regimen against new SARS-CoV-2 variants.

## Data Availability Statement

The datasets presented in this study can be found in online repositories. The names of the repository/repositories and accession number(s) can be found in the article/ Supplementary Material.

## Ethics Statement

The studies involving human participants were reviewed and approved by the Institutional Review Board of Princess Nourah Bint Abdul Rahman (IRB Number: 20-0457 in 2020), and after the permission from the head of the Army hospital. The patients/participants provided their written informed consent to participate in this study.

## Author Contributions

RB, MS, and ME-B: conceptualization. BA, TE-M, MS, and ME-B: data curation. TE-M, MS, and ME-B: formal analysis. BA, TE-M, MS, WE, and ME-B: investigation. MS, BE-H, and AG: methodology. MS, WE, and ME-B: visualization. BA, RB, WE, and ME-B: writing–original draft. TE-M and ME-B: writing –review and editing. All authors contributed to the article and approved the submitted version.

## Conflict of Interest

The authors declare that the research was conducted in the absence of any commercial or financial relationships that could be construed as a potential conflict of interest.

## Publisher’s Note

All claims expressed in this article are solely those of the authors and do not necessarily represent those of their affiliated organizations, or those of the publisher, the editors and the reviewers. Any product that may be evaluated in this article, or claim that may be made by its manufacturer, is not guaranteed or endorsed by the publisher.

## Footnotes

^1^ https://abiomix.com/decov

^2^ https://clades.nextstrain.org/

^3^ https://itol.embl.de/

^4^ https://blast.ncbi.nlm.nih.gov/

^5^ https://www.gisaid.org/epiflu-applications/covsurver-mutations-app/

^6^ https://pangolin.cog-uk.io/

## Supplementary Material

The Supplementary Material for this article can be found online at: https://www.frontiersin.org/articles/10.3389/fmicb. 2022.923137/full#supplementary-material

Supplementary Table S1 The data of sequences under investigation.
